# Stakeholders' perspectives on integrating point‐of‐care diagnostics into forensic death investigations in South Africa

**DOI:** 10.1111/1556-4029.70390

**Published:** 2026-06-19

**Authors:** Sebueng Ramatsokotla, Bathabile Soul, Kuhlula Maluleke, Lucinda Evert, Stefan Jansen van Vurren, Bettina Chale‐Matsau, Susan Mabotja, Evans Duah, Tivani Mashamba‐Thompson

**Affiliations:** ^1^ Centre for Development and Implementation of Point‐of‐Care Diagnostics, Faculty of Health Sciences University of Pretoria Pretoria South Africa; ^2^ Department of Family Medicine, Faculty of Health Sciences University of Pretoria Pretoria South Africa; ^3^ Department of Forensic Medicine, Faculty of Health Sciences University of Pretoria Pretoria South Africa; ^4^ School of Health Systems and Public Health, Faculty of Health Sciences University of Pretoria Pretoria South Africa; ^5^ International Committee of the Red Cross Pretoria South Africa; ^6^ Department of Forensic Medicine, Faculty of Health Sciences University of Free State Bloemfontein South Africa; ^7^ Department of Chemical Pathology, School of Medicine Sefako Makgatho Health Sciences University Pretoria South Africa; ^8^ National Health Laboratory Service South Africa

**Keywords:** forensic death investigation, nominal group technique, POC diagnostics, point‐of‐care testing, quality assurance, sub‐Saharan Africa

## Abstract

Unnatural and unexplained deaths present critical challenges to the criminal justice, medico‐legal, and public health systems. This study explored forensic professionals' perspectives on the integration of point‐of‐care (POC) diagnostics into unnatural death investigations in South Africa. A Nominal Group Technique (NGT) was conducted with eight key forensic stakeholders to identify and prioritize the potential benefits, facilitators, and barriers associated with POC implementation. Stakeholders ranked guidance for investigation and autopsy, improved turnaround times, and resource efficiency as the most significant POC diagnostic benefits. Scientific validity, quality assurance, and alignment with legal and regulatory frameworks were identified as critical facilitators, whereas technical limitations, quality management gaps, and legal uncertainties emerged as key barriers. Participants emphasized that POC diagnostics could support timely decision‐making during death scene investigations and autopsies, reduce laboratory backlogs, and improve family and community outcomes through faster case resolution. However, successful integration requires standardized protocols, validation, and appropriate training to ensure scientific and legal defensibility. These findings provide foundational evidence to inform the responsible integration of POC diagnostics into forensic death investigations, with potential benefits for justice delivery and community health in South Africa.

**Highlights**

- The forensic value of POC diagnostics in unnatural death investigation in South Africa was examined.
- An expert panel evaluated benefits, facilitators, and barriers associated with POC implementation.
- Standardized protocols, validation, and training are crucial for scientific and legal defensibility.
- POC diagnostics offer benefits for medicolegal death investigation in resource‐limited environments.

## 1 INTRODUCTION

Unnatural and unexplained deaths present critical challenges not only to criminal justice and medico‐legal systems but also to public health and community‐oriented primary care. According to South African law, all unnatural and unexplained deaths are subject to an inquest, as stated in the Inquest Act 58 of 1959 [1]. Thorough death investigations can significantly contribute to identifying and resolving public health concerns and are essential for ensuring that justice is served.

To enhance the quality and uniformity of death investigations within a nation or region, it is beneficial to understand promising innovative developments, such as incorporating point‐of‐care (POC) technologies [2]. POC diagnostic devices involve tests performed at or near patients' bedside rather than in a distant laboratory. Although these devices are primarily used in clinical settings, they are being increasingly integrated into forensic investigations [3]. Their use at death scenes and during autopsies could aid in determining the underlying and contributory factors, which may inform but do not necessarily constitute the cause of death [3]. This advancement can positively impact community well‐being by facilitating faster case resolution.

Building on the lessons from clinical practice, several studies have documented the effectiveness and adaptability of POC diagnostics across diverse healthcare contexts. For example, in sub‐Saharan Africa, POC technologies have been successfully implemented for the management of infectious diseases such as HIV, tuberculosis, and malaria, with evidence showing improved diagnostic turnaround times and linkage to treatment [4, 5, 6]. These clinical experiences highlight the potential of POC systems to strengthen diagnostic capacity in resource‐constrained settings, particularly through mobile‐linked platforms and decentralized testing models [7].

However, despite their success, challenges related to quality assurance, supply chain management, user training, and regulatory oversight continue to influence implementation outcomes [8, 9]. Clinicians and health system managers have addressed these challenges through structured quality management systems, task‐shifting strategies, and the development of context‐specific implementation frameworks [10]. These adaptive measures provide a useful framework for understanding how similar obstacles could be mitigated in forensic and medico‐legal environments, where issues of chain of custody, sample integrity, and validation are equally critical. Building on these lessons, the integration of POC diagnostics into forensic workflows can be informed by lessons in clinical standardization, capacity building, and sustainability planning.

These technologies enable on‐site testing of biological specimens and drugs, significantly reducing the time traditionally required to transport samples to a laboratory for analysis [11]. Examples of some POC tests used in forensic settings include portable ultrasound for preliminary whole body screening and postmortem assessment prior to autopsy; urine stick tests for glucose, acetone, and acetoacetate; blood glucose monitoring devices for measuring glucose and beta‐hydroxybutyrate levels; and the HbA1c test for measuring uncontrolled diabetes mellitus (DM) in postmortem cases [12, 13, 14]. As forensic science continues to develop, the increased adoption of POC diagnostics is expected to enhance the efficiency and responsiveness of medico‐legal investigations [3].

In the context of death scene investigations, the initial hours after a death/crime are critical for law enforcement and forensic analysis [15]. This period is crucial for gathering information on potential suspects and collecting samples and data. Therefore, the application of POC technologies in forensic science, particularly in death scene investigations, offers a means of expediting the criminal justice process by mitigating the delays associated with the conventional practice of sending evidence to forensic laboratories for examination. This approach not only accelerates the pace of investigation but also enhances the overall effectiveness of the criminal‐justice system. This study aims to evaluate the perspectives and experiences of forensic professionals regarding the implementation of POC diagnostics in unnatural death investigations. This approach seeks to assess the potential benefits and challenges associated with integrating POC diagnostics into forensic workflows, with a focus on improving the efficiency and accuracy of death investigations while contributing to broader public health initiatives. This dual‐purpose approach has the potential to strengthen both the criminal justice system and public health initiatives in South Africa.

## 2 MATERIALS AND METHODS

The Nominal Group Technique (NGT) was employed to engage key stakeholders in a structured consensus‐building process to identify and prioritize potential applications of POC diagnostics in forensic investigations, with the goal of optimizing forensic death investigations for community health purposes.

### 2.1 Study design

An NGT is a mixed‐methods approach that combines idea generation and problem‐solving in group settings [16]. It involves structured small group discussions, typically with 6–12 participants [17], though 5–9 is considered optimal [18, 19].

A distinctive feature of an NGT lies in its ability to generate diverse ideas while building consensus among participants [20]. This approach encourages active participation from all members, including those who might otherwise hesitate to share their views [21]. By promoting inclusivity, an NGT prevents dominant voices from overshadowing the knowledge creation process, ensuring a broad range of responses.

A typical NGT is through a structured process which is implemented in 4 phases.

- Silent generation of ideas: stakeholders first independently generate and document ideas in response to posed questions.
- Round‐robin sharing: This is followed by a round‐robin sharing phase, where each stakeholder shares their responses without discussion or critique, and all responses are systematically recorded.
- Discussion phase: A discussion phase allows for clarification, elaboration, and consolidation of similar ideas, with potential removal of duplicates.
- Voting phase: Finally, the stakeholders rank the listed items using a 1–7 Likert scale, where 1 indicates least important and 7 indicates most important. This ranking process results in a consolidated list of ranked themes.

### 2.2 Study setting

The NGT session was conducted online via Microsoft Teams and included key forensic stakeholders from various institutions and companies across South Africa. The online format enabled broad participation and facilitated engagement across disciplines relevant to forensic science and diagnostic practice.

### 2.3 Study population

Key stakeholders for this study were defined as individuals with experience, expertise, or active involvement in the investigation of unnatural deaths. The study population comprised forensic healthcare professionals, including forensic pathologists, forensic toxicologists, chemical pathologists, and forensic specialists who are directly involved in the delivery of medico‐legal and forensic services.

### 2.4 Sampling

Purposive sampling was employed to ensure adequate representation of relevant stakeholders. The sample size of eight participants was determined through careful consideration of the team's expertise and representation of diverse perspectives pertinent to the research question. In establishing the minimum number of participants for the NGT session, recommendations were drawn from existing literature that considered a group size of 5–9 optimal [18, 19]. Moreover, the preferred group size falls within the range of 6–12 participants, as advocated by Harvey and Holmes [22].

#### 2.4.1 Inclusion criteria

The NGT included participants who met the following criteria:

- Key stakeholders involved in unnatural death investigations.
- Individuals with expert knowledge in forensic unnatural death investigation.

#### 2.4.2 Exclusion criteria

The NGT excluded participants based on the following criteria:

- Individuals lacking expertise or experience in unnatural death investigation.
- Participants outside the specified stakeholder categories.
- Participants who refused to provide informed consent.

#### 2.4.3 Recruitment strategy

Invitations were sent via email to the identified participants, outlining the purpose of the stakeholder engagement and requesting informed consent to participate in the NGT.

### 2.5 Nominal group technique process

We conducted an online NGT session on the 11th of September 2025 by collaborating with consenting stakeholders involved in and with knowledge of unnatural death investigation. In total, eight participants were involved in the NGT session. The NGT was conducted to address three questions. The stakeholders were asked the following questions:

Question 1: How can POC be beneficial during an unnatural death investigation?

Question 2: What facilitators do you believe are present in integrating POC in unnatural death investigation?

Question 3: What are the barriers you believe are present in integrating POC diagnostics as part of unnatural death investigation?

The stakeholders were requested to answer the three questions and share their knowledge and experience about the usage of POC diagnostics tools. The Principal Investigator (PI) instructed the stakeholders to first independently list their perceptions regarding the benefits, facilitators, and barriers of implementing POC diagnostics. This was followed by a round‐robin sharing of ideas, where each stakeholder presented their points, which were recorded and consolidated. The stakeholders contributed their insights via the Microsoft Teams' meeting chat box. Subsequently, overlapping ideas were grouped into themes, and participants were given the opportunity to clarify and elaborate on points where necessary. Finally, the emerging themes were ranked anonymously using a Google Form with a 7‐point Likert scale (1 = least important, 7 = most important). The results were presented live at the end of the session. Following the NGT session, the PI compiled a report of the NGT proceedings and shared it with the stakeholders for any additional comments and feedback.

### 2.6 Data management and analysis

The study utilized a mixed‐methods approach, combining quantitative and qualitative data collection techniques. To ensure scientific rigor and facilitate comprehensive analysis, specific tools were employed for each data type. The findings were subsequently integrated to provide answers to the research inquiries.

#### 2.6.1 Quantitative data

The quantitative component of the analysis utilized Microsoft Excel spreadsheets for systematic organization and management of numerical data obtained from nominal group discussions. This structured approach facilitated statistical analysis, pattern identification, and trend detection within stakeholder responses [16]. The quantitative data primarily consisted of stakeholder‐assigned rankings for various benefits, facilitators, and barriers.

#### 2.6.2 Qualitative data

During the discussions, qualitative data were documented using the Microsoft Teams' platform. This dataset facilitated analysis, interpretation, and identification of certain themes. The information encompassed the stakeholders' ideas, comments, and discussions. One stakeholder provided additional comments in response to the session report. These insights enhanced analysis and captured additional perspectives.

Respondents evaluated the priority of each question using a 7‐point scale, ranging from 1 (least important) to 7 (most important). These individual ratings were aggregated to compute overall importance scores for all questions. Following the identification of benefits in question 1, facilitators in question 2, and barriers in question 3, all the themes addressed for each question were ranked using a similar 7‐point scale. The effectiveness ranking of each question was determined by combining scores from all the stakeholders.

### 2.7 Ethical considerations

Ethical approval for this study was obtained from the University of Pretoria Faculty of Health Sciences Research Ethics Committee (Reference No: 345/2025, dated 25 June 2025). Participants were fully informed about the background of the study, aims, and methods before their involvement. The researcher addressed any questions or concerns raised. Informed consent was obtained through signed forms, indicating voluntary participation in the NGT session. To ensure confidentiality, participants' identities and personal information were securely stored, and all discussion data were anonymized. Additionally, recorded sessions were password‐protected and encrypted to maintain security and confidentiality.

## 3 RESULTS

Eight stakeholders participated in the NGT session, contributing diverse expertise in the field of unnatural death investigation. The cohort comprised forensic pathologists, forensic specialists, a forensic genetic specialist, and chemical pathologists. This variety of perspectives and experiences enriched the discussions, ensuring comprehensive consideration of both field and laboratory incorporation of POC diagnostics. Additional characteristics, along with the specific roles of the participants, are presented in Table 1.

**TABLE 1:** NGT participants.

Stakeholder designation	Institution
Molecular Geneticist	Ampath
Forensic Toxicologist	University of Cape Town
Forensic Specialist	International Committee of the Red Cross (ICRC)
Forensic Toxicologist	Western Cape (Scientific Manager)
Forensic Pathologist	University of Free State
Forensic Genetics Specialist	African Forensic Sciences Academy (AFSA)
Forensic Pathologist	University of Pretoria
Chemical Pathologist	Sefako Makgatho Health Sciences University

### 3.1 Benefits for POC usage during unnatural death investigation

The key stakeholders identified several benefits of POC diagnostics during unnatural death investigations, which were categorized into seven themes for analysis. The identified benefits and their ranking scores are presented in ascending order of importance in Table 2.

**TABLE 2:** Ranking scores for question 1.

Benefits for POC integration	Summing by ranking votes where *1* = *low priority* and *7* = *high priority*							Total number of voting scores (number of votes × ranking score)	Percentage of votes.
	1	2	3	4	5	6	7	56	100%
Guidance for investigation and autopsy				2		1	5	49	88%
Resource efficiency and Cost‐effectiveness					2	4	2	48	85%
Speed and turnaround time			1	1	3	1	2	42	75%
Family and Community Impact			2		2	2	2	42	75%
Workforce and accessibility		2	1	1	1	3		38	68%
Contribution to Legal and Judicial Processes	1	1		3		1	2	35	63%
Advanced Applications and Innovation	2	1		2	1	2		29	52%

As shown in Table 2, stakeholders ranked guidance for investigation and autopsy as the most valuable benefit (49 points), followed closely by resource efficiency and cost‐effectiveness (48 points). Speed and turnaround time, along with family and community impact, tied for the third position (42 points each). Workforce and accessibility (38 points), contribution to legal and judicial processes (35 points), and advanced applications and innovations (29 points) were ranked lower in priority.

During the session, the stakeholders emphasized that POC diagnostics could significantly improve turnaround times and enhance efficiency in unnatural death investigations. Rapid, on‐site testing was perceived to reduce delays in toxicological and biochemical analysis and to provide preliminary results that could guide autopsy procedures and specimen collection. Stakeholders highlighted potential applications of POC tools in toxicology, genetic identification, and scene investigations. Several stakeholders noted that real‐time toxicological screening could help determine whether cases warrant further investigation, which could assist with timely decision‐making during investigations.

At the scene, a quick toxicology or blood alcohol test could help indicate whether a case should be opened or not.

The community impact of delayed laboratory results was also discussed, emphasizing that prolonged waiting periods often prolong closure for families. Rapid testing was viewed as beneficial for both the justice system and public health, enabling faster conclusions and timely burials. One of the stakeholders noted that:

Families wait forever to know exactly what happened to their loved ones… point‐of‐care will be very useful in speeding up investigations.

Families need to have answers quickly, so they are able to bury their loved one… waiting forever is very painful.

Stakeholders agreed that the introduction of POC testing could significantly reduce delays in toxicological and biochemical analysis, thereby expediting investigations and reducing case backlogs. This improvement was also perceived as a way to alleviate the emotional stress experienced by families awaiting results.

For point‐of‐care… if it's introduced in these cases, it is going to be very useful in terms of investigations being done quickly… most tests take forever, sometimes even years to conclude the case.

POC diagnostics were further described as valuable tools for guiding both scene investigations and autopsy procedures. Some stakeholders highlighted their potential role in determining which cases should proceed with full laboratory analysis:

At the scene, POC can be used as a screening tool to indicate which cases should be investigated and which cases might not have to be…postmortem, it can guide whether formal toxicology should be sent.

Stakeholders also highlighted the potential of POC diagnostics to reduce reliance on overburdened central laboratories, thereby saving time and resources. Rapid screening could help prevent unnecessary full laboratory analysis in certain cases, optimizing resource allocation. Several participants underscored the potential usefulness of POC diagnostics in resource‐limited settings, where laboratory capacity is often constrained.

In under‐resourced environments… validated point‐of‐care devices could have very good utilization in our settings.

While stakeholders acknowledged that POC results may not always be admissible in court, they stressed that rapid preliminary findings could still inform medico‐legal decision‐making and case management.

From a medico‐legal point of view, screening might be enough in cases where the poison is highly fatal.

Finally, stakeholders discussed the possibility of more advanced applications such as genetic profiling, proteomics, and toxicology screening conducted directly at the scene or mortuary.

### 3.2 Facilitators for integrating POC in unnatural death investigation

The stakeholders identified several facilitators for the integration of POC diagnostics into unnatural death investigations. These were categorized into seven themes for analysis. The identified facilitators and their ranking scores are presented in ascending order of importance in Table 3.

**TABLE 3:** Ranking scores for question 2.

Facilitators for POC integration	Summing by ranking votes where *1* = *low priority* and *7* = *high priority*							Total number of voting scores (number of votes × ranking score)	Percentage of votes
	1	2	3	4	5	6	7	56	100%
Scientific validity and reliability							8	56	100%
Legal and regulatory framework						6	2	50	89%
Quality assurance, standardization and device management			1		1	2	4	48	86%
Training and competency of personnel		1	1	1		4	1	40	71%
Cost and resource consideration		1		1	2	3	1	41	73%
Stakeholder acceptance and integration	1		1	1	2	2	1	37	66%
Operational and logistical consideration	1					6	1	37	66%

As shown in Table 3, stakeholders identified and ranked potential facilitators for integrating POC devices in unnatural death investigations. Scientific validity and reliability emerged as the most critical factor, with unanimous agreement (100%) among participants. Legal and regulatory framework followed closely (50 points), while quality assurance, standardization, and device management ranked third (48 points). Training and competency of personnel (40 points), cost and resource considerations (41 points), stakeholder acceptance and integration (37 points), and operational and logistical considerations (37 points) were also identified as significant facilitators.

The integration of POC diagnostics into forensic death investigations requires careful consideration of several enabling factors, as identified by key stakeholders. Paramount among these is the alignment with existing legislative and regulatory frameworks. Given that unnatural deaths are subject to medico‐legal investigation as stated in the Inquest Act No. 58 of 1959, stakeholders emphasized the necessity of ensuring that POC testing complies with these legal mandates.

We have to remember that unnatural deaths are an inquest… they fall within the mandate of the police.

The intended purpose of POC devices, whether for screening, diagnostic, or evidentiary use was recognized as a determinant of its legal acceptability, potentially necessitating policy and legislative revisions. Scientific reliability and quality assurance were unanimously deemed non‐negotiable. Stakeholders stressed the importance of validating the sensitivity and specificity of POC diagnostics and adhering to standardized quality‐control procedures. One participant noted:

It is not going to be helpful to pick a point‐of‐care device without knowing the specificity and sensitivity… or consistently checking if it still meets the quality.

Another highlighted the challenges in result interpretation, emphasizing the need for competency.

It's important not only for the training and competency of use, but then also the training and competency in interpreting what the result actually means and the limitations behind that result. And that could be quite challenging to disseminate.

These considerations underscore the necessity for clear standard operating procedures, regular calibration, and proficiency testing to ensure the credibility of POC generated results. Training and competency development were identified as crucial components. Users must be proficient not only in device operation but also in understanding result limitations and interpretation. This comprehensive training was viewed as essential to prevent misinterpretation and misuse, particularly in legal contexts.

Stakeholders also raised concerns regarding cost, resource allocation, and sustainability. They cautioned against reliance on donor‐funded initiatives or pilot projects, noting the challenges in maintaining POC technologies once external support ceases. As one stakeholder explained:

They would reach out to an international organization… once that funding is withdrawn, they are not able to sustain this indefinitely.

Ongoing costs related to maintenance, device procurement, reagent supply, and staff training were identified as persistent challenges requiring careful consideration during planning and implementation phases.

Lastly, stakeholder acceptance and inter‐professional collaboration were emphasized as critical for successful integration. The stakeholders stressed the importance of shared accountability and formal recognition of POC results across forensic pathology, law enforcement, laboratory specialties, and judicial authorities.

### 3.3 Barriers for integrating POC diagnostics as part of unnatural death investigation

The stakeholders identified several barriers they believed are present in implementing integrated POC diagnostics as part of unnatural death investigations. These identified barriers and their ranking scores are presented in Table 4.

**TABLE 4:** Ranking scores for question 3.

Barriers for POC integration	Summing by ranking votes where *1* = *low priority* and *7* = *high priority*							Total number of voting scores (number of votes × ranking score)	Percentage of votes
	1	2	3	4	5	6	7	56	100%
Scientific and Technical Limitations					2		6	52	93%
Quality Assurance and Protocol gaps			1		2	1	4	47	84%
Legal and Judicial Constraints			2			4	2	44	79%
Operational and Interpretation Challenges			1	1	3	1	2	42	75%
Stakeholder resistance and acceptance		1	1	2	1	1	2	38	68%
Financial and Resource Barriers		1		1	4	2		38	68%

Scientific and technical limitations emerged as the primary obstacle, receiving the highest priority score of 52. Quality assurance and protocol gaps ranked second with a score of 47, followed closely by legal and judicial constraints, which scored 44. Operational and interpretation challenges were also noted as significant barriers (42 points). Stakeholder resistance and acceptance, along with financial resource constraints, were ranked as the least pressing issues, both receiving scores of 38. These findings highlight the multifaceted nature of barriers impeding the integration of POC diagnostics in this field.

When discussing barriers to integration, stakeholders identified a range of interconnected technical, legal, and operational challenges that could impede the adoption of POC diagnostics in forensic investigations. A primary concern was the lack of standardization and technical validation across POC devices.

POC devices are introduced without proper verification… it is not going to be helpful to pick a point‐of‐care device without knowing the specificity and sensitivity, or without actually being able to consistently check to see if it is still meeting quality standards.

The variability in device specifications further underscores the need for standardized testing protocols and validation before results can be considered admissible or scientifically credible.

Legal and judicial barriers were discussed, particularly regarding the admissibility of POC results in court. The stakeholders emphasized that the legal implications depend largely on the intended purpose of testing.

Evidentiary purposes will change everything… if the point‐of‐care is used for evidentiary purposes, then that will be up to the courts to decide if they accept those results or not.

Concerns were raised that without formal legislative recognition, POC findings might face challenges in court.

Breathalyzer results have been questioned in court about quality assurance, validity, and reliability.

These observations highlight the importance of policy development and clear forensic guidelines to support the lawful integration of POC diagnostics. The absence of formal quality assurance mechanisms and operational protocols was identified as a major barrier to reliability. Stakeholders emphasized the need for standardized procedures for calibration, internal quality checks, and documentation, calling for quality management systems to govern POC deployment in forensic contexts. Operational and interpretive challenges further complicate the integration process. The ambiguous definition and scope of POC diagnostics often result in inconsistent interpretation across facilities, suggesting the need for national guidance on device selection, sample types, and reporting practices.

Resistance and limited awareness among practitioners were also identified as barriers. The stakeholders acknowledged that the concept of POC testing remains uncertain. This resistance was raised due to lack of exposure, limited training, and uncertainty about the credibility of POC data in forensic settings. Financial and resource limitations were viewed as ongoing challenges. Stakeholders emphasized that introducing POC testing requires sustained investment in devices, consumables, and training.

While POC may offer value in under‐resourced environments, it doesn't eliminate the limitations… we have to be aware that even when validated, these tests still have restrictions, and not all results can be used to determine a cause of death or stand alone as evidence.

This reflects the broader sentiment that successful integration will depend on both financial feasibility and institutional capacity to support ongoing maintenance and quality assurance.

### 3.4 Feedback from participants

The PI of the study compiled a comprehensive report of the NGT workshop, summarizing all discussions and outcomes. This report was disseminated to all eight key stakeholders for their review and feedback. Although it was assumed that all participants reviewed the document, only one participant provided additional input post‐session.

This participant emphasized the broader potential of POC diagnostics beyond chemical pathology and toxicology

POC devices have a great role to play in medicolegal death investigations. They should not be limited to chemical pathology and toxicology‐related deaths. They can also be used in microbiology or virology settings, offering significant benefits to the community. Think of a meningococcal meningitis outbreak in a highly populated environment.

In addressing barriers, the participant suggested that legislative reform is essential for effective implementation.

Changes in the legislation, including regulations, pertaining to medicolegal death investigations.

To ensure reliability and consistency, the participant also emphasized the importance of training and collaboration.

Training of healthcare providers and manufacturers of POC diagnostic devices, as well as regular review or refresher courses and collaboration between law practitioners, healthcare providers, and manufacturers or engineers to develop suitable products.

This feedback reinforced key findings from the NGT session, underscoring that successful integration of POC diagnostics in forensic death investigations will require policy reform, intersectoral collaboration, and ongoing capacity development.

## 4 DISCUSSION

This study explored forensic professionals' perspectives on integrating POC diagnostics into unnatural death investigations in South Africa. Using an NGT, a structured and fair engagement among diverse experts was achieved, leading to consensus on the perceived benefits, critical integration facilitators, and major barriers. The findings provided a contextualized understanding of how POC diagnostics can strengthen medico‐legal investigations, improve turnaround times, and ultimately enhance justice and community health outcomes.

The literature supports the reliability of POC diagnostic devices in both clinical and community settings, where they have improved early detection while maintaining acceptable accuracy [23]. However, implementation depends heavily on confidence in analytical validity and quality assurance. These concerns mirror forensic professionals' emphasis on scientific defensibility prior to courtroom acceptance.

Similarly, global adoption of POC technologies depends on credibility, standardization, and validation. Studies have shown that innovations like biosensor‐based and CRISPR‐integrated platforms achieve high sensitivity and specificity under quality frameworks, but inconsistent regulation and variable performance limit uptake [24]. Translating these lessons into forensic science underscores the necessity of a clear regulatory framework encompassing analytical validation, legal admissibility, and operator competence to ensure that POC results withstand legal scrutiny.

Global literature also emphasizes that successful implementation of molecular POC technologies must align with the World Health Organization's REASSURED criteria [25]. Integrating these principles into forensic practice could guide the development of devices and procedures that are both scientifically rigorous and operationally feasible in resource‐constrained settings.

In addition to biochemical and toxicological applications, rapid DNA technologies represent a significant advancement in molecular POC testing within forensic science [25]. These systems enable on‐site DNA profiling at the scene, mortuary, or temporary forensic facilities, with faster turnaround times, compared with traditional laboratory‐based workflows that may take days or longer. Rapid DNA technologies are increasingly utilized in disaster victim identification and mass fatality incidents, where timely identification is critical for both operational efficiency and humanitarian considerations [26, 27]. Although issues related to validation, quality assurance, and legal admissibility remain context dependent, the expanding use of rapid DNA underscores the growing role of molecular POC diagnostics in supporting time‐sensitive forensic investigations.

The South African forensic system continues to face systemic backlogs due to limited laboratory capacity, human‐resource shortages, and an increasing volume of unnatural death cases [28]. Delays in postmortem results exacerbate these backlogs, adversely affecting both the justice process and family closure. Stakeholders in this study emphasized that integrating POC diagnostics offers a promising solution by enabling rapid, decentralized, and preliminary testing to guide scene and autopsy investigations [29]. This perspective aligns with international evidence demonstrating that decentralized diagnostic models enhance service efficiency in resource‐constrained environments [11].

In clinical and public health contexts, POC technologies have proven valuable in facilitating timely decision‐making and optimizing service delivery; thus, applying similar principles in forensic practice presents an opportunity to advance both scientific accuracy and operational efficiency, while supporting community‐oriented primary care principles that promote justice and wellbeing [11].

A central theme emerging from the study is the dual role of scientific validation, reliability, and limitations as both facilitators and barriers to implementing POC diagnostics. Participants highlighted that while these technologies hold significant promise for improving efficiency, their acceptance in forensic settings depends largely on the scientific credibility of the results produced. Similar challenges have been documented in clinical settings, where successful adoption of POC testing required analytical validation, quality assurance frameworks, and comprehensive operator training to ensure reliability and reproducibility. For example, a meta‐analysis of 26 clinical studies evaluating SARS‐CoV‐2 POC tests demonstrated high pooled sensitivity (95%) and specificity (100%) when devices were validated under stringent protocols, indicating that reliability can be comparable with that of centralized laboratories with proper validation [30]. POC device evaluations have revealed that inadequate quality control, lack of external verification, and insufficient staff training can compromise POC reliability, resulting in false positive or inconclusive results.

Implementing POC diagnostics in forensic settings requires a validation framework to ensure that results are scientifically defensible and legally admissible. Regulatory frameworks in clinical diagnostics typically encompass four essential pillars: analytical validity, clinical validity, clinical utility, and regulatory compliance [4, 5, 6]. Applying a similar framework to forensic science would help ensure that devices introduced for field or autopsy use meet both scientific and legal admissibility standards. Collaboration among the Department of Health, legal professionals, and the South African Police Service (SAPS) is critical for effective data management and legal integration.

A study by Zu Y et al., [25] has emphasized that cost and sustainability remain major barriers to POC implementation, particularly in low‐ and middle‐income countries. Public and private partnerships, local manufacturing, and innovative funding models could enhance long‐term affordability and accessibility in forensic settings [24]. Moreover, effective implementation depends on skilled operators and digital connectivity through mobile or cloud‐based systems to ensure accuracy, traceability, and accountability. Integrating these approaches within forensic practice aligns with the United Nations Sustainable Development Goals specifically SDG 3 (Good Health and Wellbeing), SDG 9 (Innovation and Infrastructure), and SDG 10 (Reduced Inequalities) [31], by promoting equitable access to reliable diagnostic tools that strengthen both justice delivery and community health.

### 4.1 Strength

A significant strength of this study is its participatory design, which ensured balanced representation and prioritization of expert consensus. Notably, this is the first study of its kind to apply NGT in exploring the integration of point‐of‐care diagnostics within forensic science. The use of an NGT mitigated dominance bias and generated actionable insights suitable for translation into policy and practice. Conducting the NGT online further enhanced inclusivity, allowing participation from individuals across South Africa, irrespective of geographical location, thereby reducing travel costs. This approach not only alleviates logistical challenges but also enables the involvement of multidisciplinary experts who might otherwise be unable to attend in person. The use of Microsoft Teams helped enhance documentation and data capture. Features such as recording, chat logs, and screen sharing support accurate transcription and systematic data analysis, ensuring that no participant input is lost. The written contributions submitted through chat functions also create a verifiable audit trail, which can enhance the trustworthiness of the findings.

### 4.2 Limitations

The recruitment and participation of stakeholders posed challenges. Two participants, from the ten who consented to participate, withdrew on the day of the NGT session, reducing the number of participants to eight. Nonetheless, it is still an acceptable sample size for an NGT workshop. Additionally, connectivity issues caused delays in the commencement of the session. The study also had limited feedback from stakeholders following the distribution of the NGT workshop report, as only one participant responded. It is important to note that, as with other NGT studies, the findings are context‐specific and may not be generalizable beyond the categories represented by participating stakeholders.

### 4.3 Recommendations

Further research should incorporate perspectives from legal practitioners, law enforcement, and bereaved families to inform a more comprehensive integration model.

## 5 CONCLUSION

This study provides foundational insights into the potential sustainable integration of POC diagnostics into forensic investigations of unnatural deaths in South Africa. Stakeholders concurred that POC diagnostics could significantly and possibly enhance forensic investigations by delivering rapid, on‐site results that reduce turnaround times, guide autopsies, and alleviate laboratory backlogs. They emphasized the necessity of strong legal, operational, and scientific frameworks, standardized quality assurance, and comprehensive user training. Key challenges include technical limitations, legal uncertainties, and resource constraints. Recommendations include piloting validated POC devices with clear standard operating procedures, using them primarily as screening tools, and conducting further research on their feasibility, accuracy, and cost‐effectiveness to support broader implementation.

## CONFLICT OF INTEREST STATEMENT

The authors have no competing interests to declare.

## Data Availability

The data that support the findings of this study are available from the corresponding author upon reasonable request.
